# Mortality and risk factors of disease in Nepal: Trend and projections from 1990 to 2040

**DOI:** 10.1371/journal.pone.0243055

**Published:** 2020-12-03

**Authors:** Achyut Raj Pandey, Binaya Chalise, Niraj Shrestha, Biwesh Ojha, Jasmine Maskey, Dikshya Sharma, Peter Godwin, Krishna Kumar Aryal

**Affiliations:** 1 Nepal Health Sector Programme 3 / Monitoring, Evaluation and Operational Research, Abt Associates, Kathmandu, Nepal; 2 Graduate School for International Development and Cooperation, Hiroshima University, Hiroshima, Japan; Sciensano, BELGIUM

## Abstract

**Background:**

Between 1990 and 2017, Nepal experienced a shift in the burden of disease from communicable, maternal, neonatal and nutritional (CMNN) diseases to non-communicable diseases (NCDs). With an increasing ageing population and life-style changes including tobacco use, harmful alcohol consumption, unhealthy diets, and insufficient physical activity, the proportion of total deaths from NCDs will continue to increase. An analysis of current diseases pattern and projections of the trends informs planning of health interventions. This analysis aims to project the mortality and risk factor of disease until 2040, based on past trends.

**Methods:**

This study uses secondary data from the Global Burden of Disease (GBD) Study which analyses historic data from 1990 to 2016 to predict key variables such as, the mortality rates, life expectancy and Years of Life Lost for different causes of death from 2017 to 2040. ‘GBD Foresight Visualization’, a visualisation tool publicly available in the webpage of Institute for Health Metrics and Evaluation was the source of data for this analysis. GBD forecasting uses three-component modelling process: the first component captures variations due to risk factors and interventions, the second takes into consideration the variation due to measures of development quantified as social development index and the third uses an autoregressive integrated moving average model to capture the unexplained component correlated over time. We extracted Nepal specific data from it and reported number of deaths, mortality rates (per 100,000 population) as well as causes of death for the period 1990 to 2040.

**Results:**

In 1990, CMNN diseases were responsible for approximately two-thirds (63.6%) of total deaths in Nepal. The proportion of the deaths from the CMNN diseases has reduced to 26.8% in 2015 and is estimated to be about a fifth of the 1990 figure (12.47%) in 2040. Conversely, deaths from NCDs reflect an upward trend. NCDs claimed a third of total deaths (29.91%) in the country in 1990, while in 2015, were responsible for about two-thirds of the total deaths (63.31%). In 2040, it is predicted that NCDs will contribute to over two-thirds (78.64%) of total deaths in the country. Less than a tenth (6.49%) of the total deaths in Nepal in 1990 were associated with injuries which increased to 13.04% in 2015 but is projected to decrease to 8.89% in 2040. In 1990, metabolic risk factors including high systolic blood pressure, high total cholesterol, high fasting plasma glucose, high body mass index and impaired kidney functions collectively contributed to a tenth of the total deaths (10.38%) in Nepal, whereas, in 2040 more than a third (37.31%) of the total deaths in the country could be attributed to it.

**Conclusion:**

A reverse of the situation in 1990, NCDs are predicted to be the leading cause of deaths and metabolic risk factors are predicted to contribute to the highest proportion of deaths in 2040. NCDs could demand a major share of resources within the health sector requiring extensive multi-sectoral prevention measures, re-allocation of resources and re-organisation of the health system to cater for long-term care.

## Background

Globally, there has been a shift in the disease paradigm from communicable to non-communicable disease (NCDs) [1]. Factors like increased awareness about disease, access to water, sanitation and hygiene, cutting-edge medical treatments, vaccination and emergence of nutritional supplementations, among others, have contributed to a reduction in the burden of communicable diseases [2].

As per global estimates, by 2040, approximately four in five deaths (81%) could be due to NCDs. Meanwhile, by 2040, communicable, maternal, neonatal, and nutritional (CMNN) diseases and injurie are expected to account for approximately 12% and 7% of the global deaths respectively. Likewise, the global forecast depicts that gains in life expectancy around the world are likely to be compromised by slower gains in social demographic index (SDI); worsening condition of several risk factors including high body mass index (BMI), and stagnated gains on preventing NCDs [3].

Nepal has seen shift in disease pattern from high to low burden of infectious disease and increasing burden of NCDs [4]. Between 1990 and 2017, the life expectancy of the Nepalese population increased by 12.6 years from 58.3 to 70.9 years. In line with life expectancy, years of healthy life also increased by 11.3 years from 49.8 to 61.1 years in the same period [5]. Attributing two thirds (66%) of total deaths, NCDs are now the leading causes of death in Nepal and additional 9% of deaths are due to injuries (including unintentional injuries, transport injuries, self-harm and violence, war and disaster). CMNN diseases attribute the remaining 25% of the deaths [6].

Data on the trends of disease and associated risk factors are critical in helping to inform decision making and shape effective health interventions. This analysis aims to project the causes of mortality and risk factors of disease in Nepal from 1990 to 2040 based on secondary data of past trends.

## Methods

This study uses secondary data available from the Global Burden of Disease (GBD) Study coordinated by the Institute for Health Metrics and Evaluation (IHME) at the University of Washington. The GBD study analysed the disease burden and risk factors from over 350 diseases and injuries from 1990 onwards using more than 50,000 data sources from around the world [5, 7].

The GBD forecasting method for risk factors and disease mortality follows a three-component modelling. The first component involves computation of risk factors, prevalence and the relative risk for diseases based on risk outcome pairs [7]. It captures variations in cause of deaths due to risk factors and the interventions included in the GBD study. The basic model for cause specific mortality in the first component is specified in the equation below where *m_T_* is the total cause specific mortality, *m_U_* is the underlying mortality rate and S is the risk factor scalar (a function of all risk factors relevant to each causes included in the GBD).

$${log(m}_{T})=\log\left(m_{U}\right)+log(S)$$

The second component considers variations due to measures of development (such as income per person, education attendant and total fertility rate), which is collectively expressed in the form of social development index (SDI) in the GBD study. It captures variations in causes of deaths that were unexplained by the risk factors in the first component. It involves modelling mortality with SDI calendar time and cause specific covariates represented in the equation below where: *α_la_* is age specific intercept for specific country location, *β*_0_ and *β*_1_ captures global effect on SDI at different SDI (<0.8 and ≥ 0.8) parameters, *θ_a_t* is age specific effect on secular trend and S is risk factor scalar.

$$ŷ=\alpha_{la}+\beta_{0}{SDI}_{<0.8}+\beta_{1}{SDI}_{\geq 0.8}+\theta_{a}t+log(S)$$

The third component uses an autoregressive integrated moving average (ARIMA_1,0,0_) model to adjust variations over time that was not accounted in the first and second component, which is represented in the equation below where *ε* is the residuals representing latent trend in total cause specific mortality unexplained by risk factors, SDI and global secular trend.

$$\varepsilon ={log(m}_{T})-ŷ$$

The GBD study involves complex mathematical derivation of the equations outlined above. A detailed model specification with their derivatives is elaborated elsewhere [3].

Forecasted results are presented through GBD Foresight Visualization, a publicly accessible visualisation tool available in the webpage of IHME to allow comparison of results by age and sex across countries and their causes [8].

We extracted Nepal specific data from the GBD Foresight Visualization and reported number of deaths and mortality rates (per 100,000 population) by causes of death. GBD study categorises causes of death and their risk factors into four levels that are mutually exclusive and hierarchically nested with three broad categories at level one. Level one causes consists of CMNN diseases, NCDs, and injures. Similarly, level one risk factors include behavioural risk, metabolic risk, and environmental/occupational risk. The level one category is further classified with increasing specificity at subsequent levels. We only report level one cause to describe mortality trends until 2040. We also present data on attributable death for level one causes by all risk factors as well as by level two risk factors for all causes. We extracted data from multiple year to depict the changing trend in the mortality and risk factors and have presented them along with 95% uncertainty interval (UI).

## Results

In 1990, CMNN diseases were the leading cause of deaths, attributing approximately two in every three deaths (63.60%, 95% UI: 58.63–68.07) in Nepal. In the same period, NCDs were responsible for nearly one third of the total deaths (29.91%, 95% UI: 26.00–34.12). By 2015 the situation has reversed, NCDs became the major cause of death (63.21%, 95% UI: 59.25–66.75) and CMNN diseases causing less than a third of the total deaths (26.8%, 95% UI: 23.30–31.18). By 2040, the burden of NCDs is estimated to rise further causing close to four in five deaths (78.64%, 95% UI: 70.37–83.50) in the country. Meanwhile, the estimated total deaths due to CMNN disease in the same year would have reduced further with about one in ten deaths (12.47%, 95% UI: 7.98–21.07). Likewise, injuries which was responsible for 6.49% (5.49–7.51) of total deaths in 1990, claimed 13.04% (95% UI: 11.84–13.9) of lives in 2015 and will be responsible for 8.89% (95% UI: 7.16–10.57) of total deaths in 2040 [Table 1].

**Table 1:** Causes of deaths in Nepal.

Sex	Year	CMNN disease			NCDs			Injuries		
		Number of deaths	Death rate per 100000	Attributable deaths (%)	Number of deaths	Death rate per 100000	Attributable deaths (%)	Number of deaths	Death rate per 100000	Attributable deaths (%)
Both gender	1990	134601 (112574–156280)	717.94 (600.45–833.57)	63.6 (58.63–68.07)	63086 (55430–70342)	336.49 (295.65–375.19)	29.91 (26–34.12)	13698 (11524–16081)	73.06 (61.47–85.78)	6.49 (5.49–7.51)
	1995	117235 (101008–133669)	548.31 (472.42–625.17)	57.93 (53.02–62.48)	70254 (62157–78014)	328.58 (290.71–364.87)	34.84 (30.57–39.19)	14711 (12816–16560)	68.8 (59.94–77.45)	7.28 (6.36–8.21)
	2000	94344 (81248–107490)	397.7 (342.49–453.11)	50.01 (45.7–54.29)	78250 (71388–84856)	329.86 (300.93–357.7)	41.6 (37.75–45.32)	15882 (13908–17597)	66.95 (58.63–74.18)	8.43 (7.42–9.26)
	2005	74105 (63545–85262)	289.73 (248.45–333.36)	40.54 (36.43–44.91)	91501 (84221–98035)	357.75 (329.28–383.29)	50.19 (46.36–53.75)	17039 (14851–18863)	66.62 (58.06–73.75)	9.33 (8.17–10.18)
	2010	63578 (54384–73961)	233.85 (200.03–272.04)	35.12 (31.1–39.54)	102045 (93224–110473)	375.33 (342.89–406.33)	56.48 (52.5–60.16)	15369 (12998–17237)	56.53 (47.81–63.4)	8.49 (7.25–9.33)
	2015	50946 (43154–60556)	171.88 (145.59–204.3)	26.8 (23.3–31.18)	114291 (104512–123138)	385.58 (352.59–415.43)	63.21 (59.25–66.75)	24755 (22371–26791)	83.52 (75.47–90.39)	13.04 (11.84–13.9)
	2020	38102 (27166–58654)	121.58 (86.78–188.69)	21.28 (15.92–30.27)	123480 (105811–141035)	393.98 (337.24–452.32)	69.38 (60.67–74.71)	16582 (14091–19131)	52.91 (44.85–61.28)	9.34 (7.7–10.98)
	2025	33656 (22783–53371)	103.26 (70.1–164.74)	17.96 (12.73–27.18)	135346 (116968–152556)	415.18 (358.68–470.8)	72.61 (64.02–77.8)	17545 (14658–20064)	53.82 (45.17–61.78)	9.43 (7.73–11.1)
	2030	31382 (20409–52169)	93.45 (60.59–155.96)	15.55 (10.64–24.42)	150569 (130052–169117)	448.23 (383.07–508.96)	74.99 (66.32–79.97)	18963 (15689–22630)	56.44 (46.58–67.14)	9.47 (7.71–11.3)
	2035	30202 (18873–51619)	88.41 (54.69–151.78)	13.83 (9.09–22.31)	167206 (144222–188250)	489.27 (417.8–557.32)	76.99 (68.3–81.83)	19880 (16767–22861)	58.16 (48.73–67.39)	9.18 (7.45–10.79)
	2040	29439 (17754–51599)	85.52 (50.93–152.07)	12.47 (7.98–21.07)	184693 (158568–209511)	536.27 (454.36–616.17)	78.64 (70.37–83.5)	20816 (17119–24160)	60.42 (49.27–70.94)	8.89 (7.16–10.57)
Male	1990	63897 (52895–75512)	686.57 (568.35–811.37)	63.03 (57.67–68.36)	29008 (25105–32877)	311.69 (269.75–353.26)	28.74 (24.17–33.5)	8327 (6914–9728)	89.47 (74.29–104.52)	8.24 (6.85–9.59)
	1995	55217 (46742–64126)	511.43 (432.93–593.95)	56.49 (51.06–61.55)	33386 (29095–37655)	309.23 (269.48–348.77)	34.31 (29.83–39.07)	9047 (7747–10415)	83.8 (71.76–96.47)	9.27 (8.04–10.58)
	2000	44459 (37728–51413)	376.82 (319.78–435.76)	47.47 (42.97–52.13)	39295 (35723–42738)	333.05 (302.78–362.24)	42.1 (38.16–46.08)	9806 (8496–11130)	83.12 (72.01–94.34)	10.49 (9.12–11.81)
	2005	35109 (29386–41725)	276.94 (231.79–329.13)	38.34 (33.56–43.39)	46030 (41478–50408)	363.08 (327.18–397.62)	50.43 (46–54.52)	10350 (8823–11742)	81.64 (69.6–92.62)	11.32 (9.71–12.55)
	2010	30456 (25357–36701)	229.78 (191.31–276.9)	33.21 (28.19–39.69)	51957 (45847–58375)	392.01 (345.91–440.43)	56.77 (51.75–61.42)	9302 (7492–10830)	70.18 (56.53–81.71)	10.14 (8.32–11.45)
	2015	24201 (19588–30446)	168.07 (136.04–211.44)	24.91 (20.65–30.9)	57905 (50642–65414)	402.14 (351.71–454.29)	63.2 (58.19–67.38)	15055 (13248–16613)	104.56 (92.01–115.37)	15.51 (13.77–16.84)
	2020	17689 (12406–26793)	115.9 (81.25–175.36)	19.3 (14.21–27.8)	63464 (53648–75112)	415.83 (350.5–493.26)	69.41 (61.55–74.51)	10301 (8385–12369)	67.49 (54.86–81.28)	11.3 (9.18–13.35)
	2025	15635 (10603–24601)	98.2 (66.22–154.72)	16.21 (11.31–24.85)	69640 (59528–80478)	437.45 (371.1–507.5)	72.38 (64.42–77.38)	10949 (8942–12987)	68.78 (55.81–82.24)	11.41 (9.23–13.51)
	2030	14508 (9414–23894)	88.28 (56.84–147.26)	14.03 (9.29–22.59)	76860 (65119–87979)	467.74 (396.59–540.61)	74.53 (66.9–79.41)	11775 (9393–14505)	71.65 (57.1–88.5)	11.44 (9.14–13.94)
	2035	13921 (8696–23490)	83.16 (51.28–141.99)	12.54 (7.96–20.83)	84550 (71874–96391)	505.11 (428.15–583.32)	76.37 (68.3–81.08)	12246 (9876–14757)	73.15 (58.92–88.7)	11.09 (8.87–13.2)
	2040	13576 (7977–23965)	80.4 (47.51–143.35)	11.39 (6.89–19.58)	92637 (78719–106147)	548.6 (460.52–635.33)	77.93 (69.68–82.81)	12665 (10259–15171)	74.98 (60.23–91.2)	10.68 (8.48–12.82)
Female	1990	70704 (58691–81644)	748.87 (621.63–864.75)	64.14 (57.81–70.08)	34078 (28052–40069)	360.94 (297.11–424.39)	30.99 (25.43–36.48)	5371 (4176–6808)	56.89 (44.23–72.11)	4.88 (3.78–6.12)
	1995	62018 (52997–71597)	585.93 (500.7–676.43)	59.29 (52.72–66.15)	36869 (29697–43210)	348.32 (280.57–408.23)	35.33 (29.15–41.3)	5664 (4562–6858)	53.51 (43.1–64.79)	5.42 (4.4–6.52)
	2000	49885 (42427–57545)	418.35 (355.81–482.6)	52.53 (46.81–59.09)	38955 (32992–44381)	326.69 (276.68–372.2)	41.1 (35.18–46.07)	6075 (5089–7136)	50.95 (42.68–59.85)	6.4 (5.38–7.46)
	2005	38996 (32935–45515)	302.31 (255.32–352.84)	42.76 (37.86–48.62)	45470 (39831–50219)	352.5 (308.78–389.31)	49.93 (44.57–54.29)	6689 (5675–7680)	51.85 (43.99–59.54)	7.34 (6.27–8.34)
	2010	33122 (28072–38858)	237.71 (201.47–278.87)	37.09 (32.44–43.11)	50088 (43969–55121)	359.47 (315.56–395.59)	56.17 (50.43–60.49)	6068 (5036–6950)	43.55 (36.14–49.88)	6.8 (5.66–7.66)
	2015	26745 (22520–32149)	175.47 (147.75–210.92)	28.8 (24.74–34.36)	56386 (50336–61660)	369.93 (330.24–404.53)	63.2 (57.73–67.24)	9700 (8605–10555)	63.64 (56.45–69.25)	10.45 (9.31–11.39)
	2020	20413 (13836–33888)	126.98 (85.97–210.68)	23.38 (16.76–35.57)	60016 (49823–69959)	373.27 (308.96–436.91)	69.34 (57.98–75.78)	6281 (5149–7273)	39.06 (31.85–45.22)	7.28 (5.71–8.82)
	2025	18021 (11592–30857)	108.09 (69.25–184.13)	19.82 (13.35–31.34)	65706 (54964–75601)	393.96 (327.83–456.85)	72.84 (61.63–79.34)	6596 (5361–7600)	39.54 (31.99–45.74)	7.34 (5.69–8.95)
	2030	16874 (10364–29841)	98.4 (59.69–174.67)	17.14 (11.03–28.16)	73709 (61630–85041)	429.6 (355.62–502.05)	75.47 (64.95–81.68)	7188 (5781–8503)	41.88 (33.36–49.79)	7.39 (5.66–9.07)
	2035	16282 (9451–30070)	93.46 (54.08–174.01)	15.17 (9.36–25.83)	82656 (69024–95632)	474.13 (388.81–555.51)	77.63 (67.11–83.75)	7633 (6046–8984)	43.77 (34.34–51.79)	7.2 (5.51–8.75)
	2040	15863 (8928–30224)	90.46 (50.35–174.07)	13.57 (8.02–23.94)	92057 (75979–108267)	524.51 (422.87–623.79)	79.37 (68.94–85.33)	8151 (6289–9724)	46.42 (35.67–55.86)	7.06 (5.32–8.71)

There is a notable decline in the mortality rate of CMNN diseases from 717.94 (95% UI: 600.45–833.57) per 100,000 population in 1990 to 171.88 (95% UI: 145.59–204.3) per 100,000 population in 2015 and is estimated to decline further reaching 85.52 (95% UI: 50.93–152.07) per 100000 population by 2040. Mortality rates for NCDs however, increased from 336.49(295.65–375.19) per 100,000 population in 1990 to 385.58 (95% UI: 352.59–415.43) per 100,000 population in 2015 and is predicted to reach 536.27 (95% UI: 454.36–616.17) per 100,000 population by 2040 [Table 1].

There are sex differentials in the mortality rate with more females dying from CMNN diseases and more males losing their lives due to NCDs and injuries. In 2015, mortality rates among male was 168.07 (95% UI: 136.04–211.44) per 100,000 population for CMNN diseases, 402.14 (95% UI: 351.71–454.29) per 100,000 population for NCDs and 104.56 (95% UI: 92.01–115.37) per 100,000 population for injuries. The mortality rate among females was 175.47 (95% UI: 147.75–210.92) per 100,000 population for CMNN diseases, 369.93(95% UI: 330.24–404.53) per 100,000 population for NCDs and 63.64 (95% UI: 56.45–69.25) per 100,000 population for injuries. However, these differences are projected to narrow down by 2040. By the year 2040, the mortality rate among male is estimated to be 80.4(95% UI: 47.51–143.35) per 100,000 population for CMNN diseases, 77.93 (95% UI: 69.68–82.81) per 100,000 population for NCDs and 74.98(60.23–91.2) per 100,000 population for injuries while mortality rates for females is predicted to be 90.46 (95% UI: 50.35–174.07) per 100,000 population for CMNN diseases, 79.37 (95% UI: 68.94–85.33) per 100,000 population for NCDs and 46.42 (95% UI: 35.67–55.86) per 100,000 population for injuries [Table 1].

### Risk factors attribution

In the year 1990, around three in five deaths in the country (63.08%) were attributed to three categories of risk factors combined: behavioural, metabolic and environmental risk factors. This proportion of deaths from the risk factors is expected to decrease slightly by 2040 attributing 57.58% of total deaths. Remaining deaths are not attributable to any of these risk factors [not shown in table].

### Environmental risk factors

The proportion of total deaths from environmental risk factors is estimated to halve in 2040, with a decline to 16.54% (95% UI: 13.07–21.72) in 2040 from 32.23% (95% UI: 28.04–36.45) in 1990. The total attributable deaths due to air pollution and unsafe water, sanitation and handwashing has also declined and is likely to continue in this direction. Air pollution attributed 15.1% (95% UI: 12.89–17.48) of total deaths in 1990, 17.7% (95% UI: 15.66–19.62) of deaths in 2015 and is estimated to contribute to 10.89% (95% UI: 8.6–13.46) of total deaths in 2040. Unsafe water, sanitation, and hand washing, which were the most important environmental risk factors in 1990, resulted in 17.25% (95% UI: 12.95–22.25) of the total deaths, witnessed a four-fold reduction in 2015 (4.88%, 95% UI: 2.88–7.71)) and is estimated to halve by 2040. Occupational risk factors which were responsible for 0.85% (95% UI: 0.59–1.11) of total deaths in 1990 and are predicted to attribute 2.4% (95% UI: 1.75–3.34) in 2040. Similarly, deaths attributable to other environmental risk factors (like residential Redon and lead exposure) are also predicted to increase from 0.68% (95% UI: 0.39–1.00) to 1.92% (95% UI: 1.11–2.92) from 1990 to 2040 [Table 2].

**Table 2:** Deaths attributable to environmental risk factors (in %).

Year	Air pollution	Occupational risk	Unsafe water-sanitation-and hand washing	Other environmental risk	All environmental risks factors
1990	15.1 (12.89–17.48)	0.85 (0.59–1.11)	17.25 (12.95–22.25)	0.68 (0.39–1)	32.23 (28.04–36.45)
1995	15.25 (13.23–17.59)	1.01 (0.73–1.29)	16.75 (11.95–22.08)	0.89 (0.54–1.27)	32.24 (28.11–36.65)
2000	16.8 (14.75–19.03)	1.17 (0.86–1.47)	11.87 (8.46–16.09)	1.16 (0.73–1.64)	29.21 (25.76–32.9)
2005	18.2 (16.13–20.34)	1.44 (1.06–1.83)	8.69 (6.05–11.95)	1.45 (0.94–2.02)	27.85 (24.86–30.97)
2010	18.57 (16.46–20.63)	1.84 (1.41–2.28)	7.58 (4.91–11.02)	1.63 (1.07–2.28)	27.56 (24.48–30.81)
2015	17.7 (15.66–19.62)	2.02 (1.59–2.46)	4.88 (2.88–7.71)	1.67 (1.06–2.32)	24.32 (21.78–27.26)
2020	17.34 (14.3–20.59)	2.35 (1.7–3.34)	4.78 (1.39–13.01)	1.91 (1.15–2.77)	24.42 (20.18–30.93)
2025	15.68 (12.89–18.83)	2.41 (1.77–3.37)	4.15 (1.09–11.93)	1.95 (1.17–2.86)	22.38 (18.38–28.49)
2030	13.87 (11.23–16.8)	2.41 (1.77–3.33)	3.6 (0.84–10.79)	1.95 (1.16–2.91)	20.2 (16.35–25.92)
2035	12.18 (9.74–14.86)	2.4 (1.76–3.31)	3.12 (0.67–9.58)	1.94 (1.13–2.91)	18.16 (14.52–23.61)
2040	10.89 (8.6–13.46)	2.4 (1.75–3.34)	2.67 (0.53–8.5)	1.92 (1.11–2.92)	16.54 (13.07–21.72)

In 2015, among causes of air pollution, indoor air pollution attributed 11.36% (95% UI: 9.42–13.42) and ambient air pollution attributing to 8.82% (95% UI: 7.6–10.1) of the total deaths. The situation is predicted to change in 2040 with ambient air pollution 6.93% (5.41–8.52) of deaths being the major attributor compared to household air pollution 4.16% (95% UI: 2.83–6.05) [Not shown in table].

### Behavioural risk factors

Behavioural risk factors were the leading causes of deaths in 1990 causing 43.35% (95% UI: 39.35–47.49) of the total deaths and could be the leading cause of deaths until 2030 causing 34.33% (95% UI: 28.63–39.12) of the total deaths. With a dramatic decline in total deaths attributable to the child and maternal malnutrition from 29.43% (95% UI: 24.65–34.17) in 1990 to 0.98% (95% UI: 0.56–1.52) in 2040, the proportion of total deaths from all behavioural risk factors combined declined from 43.35% (95% UI: 39.35–7.49) in 1990 to 37.86% (95% UI: 35.09–40.71) in 2015. Moreover, the downward trend is likely to continue and reach 31.71% (95% UI: 25.84–37.05) in 2040.

Alcohol and drug use, dietary risk, low physical activity, tobacco use, and sexual violence likely contribute to a higher proportion of deaths in 32040 compared to 1990. Total deaths attributable to alcohol and drug use is projected to increase from 0.7% (95% UI: 0.54–0.89) to 3.24% (95% UI: 2.23–4.67), dietary risk from 7.06% (95% UI: 5.65–8.91) to 18.23% (95% UI: 12.54–24.3), low level of physical activity from 0.6% (95% UI: 0.3–0.97) to 2.33% (1.14, 4.06), tobacco use from 9.15% (95% UI: 7.53–10.9) to 12.35% (10.18, 14.6) and sexual violence from 0.01% (95% UI: 0.00–0.01) to 0.02% (95% UI: 0.01–0.03) from 1990 to 2040, according to the projections [Table 3].

**Table 3:** Deaths attributable to behavioural risk factors (in %).

Year	Alcohol and drug use	Child and maternal malnutrition	Dietary risk	Low physical activity	Tobacco use	Sexual violence	All behavioural risk factors
1990	0.7 (0.54–0.89)	29.43 (24.65–34.17)	7.06 (5.65–8.91)	0.6 (0.3–0.97)	9.15 (7.53–10.9)	0.01 (0–0.01)	43.35 (39.35–47.49)
1995	1.1 (0.84–1.4)	25.76 (21.89–29.74)	8.43 (6.88–10.23)	0.76 (0.38–1.21)	10.4 (8.77–12.24)	0.01 (0.01–0.02)	42.2 (38.59–45.73)
2000	1.86 (1.46–2.35)	21.88 (18.56–25.41)	10.65 (8.91–12.74)	1.03 (0.52–1.65)	12.3 (10.57–14.1)	0.01 (0.01–0.02)	42.2 (38.77–45.05)
2005	2.39 (1.82–3.07)	15.74 (12.94–18.61)	13.9 (11.43–16.33)	1.38 (0.7–2.19)	14.76 (12.9–16.56)	0.03 (0.02–0.05)	41.19 (38.44–43.69)
2010	2.6 (1.96–3.34)	12.08 (9.97–14.26)	16.38 (13.58–19.07)	1.68 (0.85–2.66)	15.45 (13.49–17.17)	0.03 (0.02–0.05)	40.63 (37.79–43.39)
2015	2.68 (2.05–3.46)	8.51 (6.59–10.71)	17.45 (14.45–20.41)	1.85 (0.96–2.9)	14.24 (12.35–15.95)	0.03 (0.02–0.04)	37.86 (35.09–40.71)
2020	3.03 (2.08–4.25)	4.52 (3.05–6.3)	19.7 (14.94–24.08)	2.18 (1.08–3.58)	14.89 (12.33–17.18)	0.03 (0.02–0.04)	37.17 (31.79–41.36)
2025	3.13 (2.16–4.41)	2.98 (1.94–4.33)	19.77 (14.77-A,24.33)	2.29 (1.14–3.78)	14.42 (11.91–16.7)	0.02 (0.01–0.04)	35.77 (30.29–40.16)
2030	3.16 (2.18–4.48)	2 (1.25–2.99)	19.45 (14.21–24.53)	2.34 (1.16–3.95)	13.76 (11.34–16.07)	0.02 (0.01–0.04)	34.33 (28.63–39.12)
2035	3.2 (2.24–4.59)	1.4 (0.84–2.14)	18.9 (13.31–24.54)	2.36 (1.16–4.09)	13.03 (10.76–15.27)	0.02 (0.01–0.03)	33 (27.14–37.9)
2040	3.24 (2.23–4.67)	0.98 (0.56–1.52)	18.23 (12.54–24.3)	2.33 (1.14–4.06)	12.35 (10.18–14.6)	0.02 (0.01–0.03)	31.71 (25.84–37.05)

### Metabolic risk factors

Metabolic risk factors like high systolic blood pressure (BP), high total cholesterol, high fasting plasma glucose, high BMI and impaired kidney functions, will be the only broad category of risk factor that will have increase share in total deaths according to the projections. Metabolic risk factors were responsible for 10.38% (95% UI: 8.72–12.39) of deaths in 1990 and are projected to be responsible for 37.31% (95% UI: 28.86–44.93) of deaths in 2040. Attribution of high BMI could increase by almost double from 5.28% (95% UI: 2.66–8.41) in 2015 to 11.36% (95% UI: 6.08–17.55) in 2040. Similarly, the attribution of raised fasting plasma glucose is projected to increase from 8.68% (95% UI: 6.71–11.12) in 2015 to 15.52% (95% UI: 10.96–20.29) in 2040, and impaired kidney functions from 4.57% (95% UI: 3.94–5.2) in 2015 to 8.11% (95% UI: 5.28–12.91) in 2040 [Table 4].

**Table 4:** Deaths attributable to metabolic risk factors (in %).

Year	Raised systolic BP	High total cholesterol	Fasting plasma glucose	High BMI	Impaired kidney function	All metabolic risk factors
1990	7.07 (5.79–8.48)	2.11 (1.53–2.79)	2.91 (2.09–3.94)	1.01 (0.4–1.98)	2.02 (1.69–2.45)	10.38 (8.72–12.39)
1995	8.59 (7.16–10.13)	2.7 (1.98–3.58)	3.69 (2.7–4.89)	1.53 (0.66–2.74)	2.41 (2.04–2.86)	16.17 (14.27–18.2)
2000	10.83 (9.34–12.43)	3.73 (2.75–4.84)	4.81 (3.55–6.34)	2.14 (0.97–3.66)	3 (2.58–3.5)	20.69 (18.52–22.84)
2005	13.75 (12.01–15.5)	4.95 (3.74–6.28)	6.33 (4.78–8.22)	3.02 (1.38–5.03)	3.9 (3.37–4.44)	24.23 (21.7–26.69)
2010	15.96 (14–18.05)	5.85 (4.41–7.45)	7.53 (5.72–9.69)	4.04 (1.94–6.54)	4.57 (3.94–5.2)	26.6 (23.93–29.14)
2015	17.21 (15.03–19.22)	6.43 (4.86–8.25)	8.68 (6.71–11.12)	5.28 (2.66–8.41)	4.99 (4.27–5.7)	31.52 (24.81–36.9)
2020	20.02 (15.42–24.02)	7.31 (5.1–9.95)	10.87 (7.86–14.16)	6.95 (3.54–11.08)	6.08 (4.51–8.2)	12.74 (10.86–14.92)
2025	20.71 (15.87–25.2)	7.46 (5.06–10.43)	12.16 (8.77–15.84)	7.96 (4.1–12.63)	6.6 (4.78–9.26)	33.43 (26.02–39.22)
2030	21.09 (16.05–26.3)	7.48 (4.94–10.81)	13.38 (9.62–17.42)	8.99 (4.67–14.08)	7.09 (4.94–10.41)	34.96 (27.3–41.36)
2035	21.28 (15.93–27.01)	7.4 (4.71–10.82)	14.51 (10.29–18.92)	10.11 (5.32–15.72)	7.58 (5.18–11.64)	36.25 (28.23–43.03)
2040	21.37 (15.77–27.51)	7.21 (4.43–10.81)	15.52 (10.96–20.29)	11.36 (6.08–17.55)	8.11 ((5.28–12.91)	37.31 (28.86–44.93)

## Discussion

Nepal is currently facing an increasing burden of NCDs. However, in 1990 CMNN diseases were the leading cause of deaths accounting for close to two-thirds of the total deaths in the country (63.6%), while NCDs claimed a third (29.91%). However, in 2015, there was a monumental shift. By this time, NCDs were the leading cause of deaths in the country, claiming 63.21% of the total deaths and CMNN diseases were responsible for 26.8% of the total deaths. Going forward, in 2040, four out of every five deaths will be due to NCDs (78.64%) while CMNN diseases will cause just over a tenth of all the deaths (12.47%) according to the projections. Injuries, which were responsible for 6.49% of total deaths in 1990, claimed 13.04% of lives in 2015 and will be responsible for 8.89% of total deaths in 2040 according to projections.

NCDs have also become the leading cause of deaths in other neighbouring countries of Nepal like Bangladesh, Pakistan, and Bhutan. In 2015, NCDs were responsible for 69.26% of deaths in Bangladesh, 68.3% of deaths in Bhutan and 62.47% deaths in Pakistan. Similarly, CMNN diseases were responsible for 22.03% of deaths in Bangladesh, 22.81% of deaths in Bhutan, and 30.58% deaths in Pakistan. And again, the proportion of deaths due to injuries was 8.7% in Bangladesh, 8.57% in Bhutan and 7%% in Pakistan, in the year 2015 [8]. In India, 27·5% of deaths were due to CMNN diseases, 61·8% of deaths were due to NCDs, and 10·7% due to injuries in 2016. These data are comparable to the proportion of deaths due to CMNN diseases, NCDs and injuries in Nepal for the year 2015 [9]. The changes between 1990 to 2015 in these countries is similar to that of Nepal [8, 9].

Factors like an ageing population, increase in urban population with sedentary lifestyle, changes in diet pattern with increased consumption of junk foods, alcohol and tobacco consumption could be responsible for the increase in the burden of NCDs. With this increasing burden, NCDs have received more policy attention in recent years. Nepal has demonstrated a significant progress in reducing the mortality due to CMNN diseases. The declining burden of communicable diseases may be attributable in part to disease-specific priority health interventions such as the diarrhoea, malaria, leishmaniasis and tuberculosis control programmes. Increased coverage of immunisation services could be the reasons for reduction in mortality due to measles tetanus and other infectious disease. Maternal health has been a priority area for the government of Nepal with a series of policy initiatives like maternity incentive schemes, skilled births attendant policy, birth preparedness package, and expansion of family planning services which could be responsible for the reduction in maternal mortality. For example, the percentage of mothers who received four ANC consultations increased from 14% in 2001 to 69% in 2016 and the institutional delivery rate increased from 8% in 1996 to 57% in 2016 [10].

Among three broad categories of risk factors of NCDs (metabolic, behavioural and environmental), metabolic risk factors will be the only risk factors whose share of the total deaths will have increased from 2015 to 2040 according to the projections. Attribution of a high BMI raised fasting plasma glucose, and impaired kidney functions may almost double from 2015 to 2040. Furthermore, the attribution of raised systolic BP and high total cholesterol could also increase from 2015 to 2040. Unsafe water was a major environmental risk factor in 1990 whereas air pollution will be the major risk factor 2040 hinting towards a much needed additional focus in reducing the exposure to indoor and ambient air pollution.

Acknowledging the changing burden of disease, the Nepal Health Sector Strategy 2015–2020 had outlined NCD specific targets including a reduction in the prevalence of raised blood pressure from 25.7% to 22% as well as the reduction in the prevalence of tobacco use among 15–29 years age group from 11.4% to 9.2% between 2013 to 2020 [11]. Although the government of Nepal developed a Multisectoral Action Plan for the Prevention and Control of NCDs (2014–2020) and has introduced Packages of Essential NCDs Interventions (PEN) into primary health care system in a phase wise approach; the lack of supply side capacity to efficiently manage NCDs have not yielded major results [12]. Interventions such as the control of tobacco products and alcohol use, standardised protocol for management of chronic diseases and a surveillance system are some of the important government initiatives although their effectiveness is yet to be documented [13, 14].

The institutions offering basic health care are a critical touchpoint to implement government interventions. Although NCDs are the leading cause of mortality, medicines for NCDs, including cardiovascular diseases are not universally available in the Nepalese public health facilities. A nationwide survey in 2015 revealed the atenolol was available in 18% of health facilities (HFs), amlodipine was available in 11% of HFs and aspirin was available in 10% of the HFs. Treatment of NCDs will demand a re-orientation of health care services themselves, towards, longer-term, chronic care models. The repositioning will require realignment of the human resource including differentiated basic and follow-on training, task-shifting and reassignment of tasks.

Despite the high burden, Nepal’s fiscal strategy places a low priority on NCDs and injuries (NCDIs)–either for prevention or for care. In 2014, 6.4% of the total government expenditure on health was for NCDIs [15, 16]. With a weak and underfunded health system, most of the countries in LMICs are undergoing a protracted epidemiological transition [17, 18], a condition where NCDs are in increasing trend still with a notable presence of CMNN diseases. The competing priorities cause a dilemma about which services to include or exclude from the list of essential health services that are delivered free of cost to the entire population [18, 19]. Meanwhile, in the absence of regular and preventive health check-ups, NCDs tends to go undetected and manifest at a later stage which increases the cost of disease management [20]. Epidemiological burden together with rapid population ageing in countries like Nepal can pose exceptionally serious sustainability challenges. Appropriate health financing arrangements with systematic and transparent priority setting process can ensure availability of resources for the right services at the right time [21].

NCDs pose a complex challenge. They require proactive measures on multiple fronts, including risk factor prevention, health promotion, and clinical care and management. NCDs are often associated with a lifestyle where individual choices and the environment complementing those choices play a crucial part. Besides promoting the importance of healthy lifestyles, policies and strategies need to focus on the creation of an enabling environment or an ecosystem where lifestyle modification is achievable. Behaviour change depends on factors like the cost of processed food versus fresh produce, distance to the fruit and vegetable markets or a place for exercise [22]. Consumer’s choices are often shaped by an “obesogenic environment” [23] where our daily foods are considered as means of satiety influenced by social and cultural circumstances [22]. Having functioning dietary guideline can be useful in achieving effective change in dietary behaviour at a consumer’s level [24]. A study has concluded that dietary policies in Nepal do not match the burden of disease, and the government actions are not designed to adequately target critical dietary drivers [25]. According to Popkin’s framework, Nepal is currently in the fourth stage of nutrition transition [26]. Per-capita energy consumption from fat has doubled, and sugar and sweeteners consumption has witnessed a nine-fold increase per capita between 1970 and 2010 [26]. As of 2017, dietary risks, high blood pressure, high fasting plasma glucose, and high body mass index occupy the third, fifth, sixth and ninth position respectively, in the top ten risk factors that contribute to disability adjusted life years (DALYs) in Nepal [27]. These facts, in part, explains the cause of this reversal in disease burden in the last thirty years. Therefore, it is essential to understand the factor affecting the dietary choices of the Nepalese population to design a holistic approach to combat the NCDs.

A previous Cochrane review had concluded that the combination of multi-pronged strategies is the most effective interventions for improvements in chronic disease management [28]. A recent version of a Chronic Care Model also has advocated the integration of population health promotion with clinical service delivery. Broader, interdisciplinary, and inclusive teams working collaboratively and with the community support and leadership can be useful in overcoming barriers in the utilisation of services and preventing diseases. By strategically focusing on both the prevention and treatment as a continuum, Nepal has the best potential for improving health outcomes [29].

Taxation on tobacco and alcohol products could also be a useful strategy to reduce consumption apart from generating revenue. The deaths attributable to tobacco and alcohol consumption has also increased from the year 1990 to 2015. Prevalence of tobacco smoking (including cigarettes, pipes, cigars or any other smoked tobacco products, both daily and non-daily smokers) was 23.8% in 2007 [30], 18% in 2013 [31] and 17% in 2019 [32] which indicate a marginal decline in the rates. Apart from taxation, evidence suggest that other preventive strategies like warning signals on tobacco package, counselling for quitting smoking and alcohol use through health workers could be useful in reducing the prevalence of risk factors. Around 44.8% of current smokers thought about quitting because of warning labels on cigarette packages. Approximately 19.4% had tried to stop smoking while around 22.1% of smokers reported being advised by a health care provider to stop smoking/use of smokeless tobacco in the last 12 months [32].

Similarly, the prevalence of being a current drinker (those who consumed any alcohol within the past 30 days) was 28.5% in 2007 [30], 17.4% in 2013 [31] and 23.9% in 2019 [33] depicting fluctuating trend in alcohol consumption. In recent a NCDs risk factors STEPS survey, around 6.8% of adults in Nepal were found to be engaged in heavy episodic drinking (consumption of at least six standard drinks or 60g of pure alcohol on a single occasion). Alcohol consumption is also an important factor in road traffic accidents and injuries. In the same study, almost 17.2% had driven a vehicle under the influence of alcohol in the past 30 days. Similarly, 8.4% of adults had ridden in a motorised vehicle with the driver had two or more alcoholic drinks [33]. The government of Nepal aims to strengthen the enforcement and compliance to tobacco product control and regulatory provisions as the core strategy in reducing tobacco consumption combined with improved public awareness. The government also has the strategy to reduce public availability of alcohol products and raise awareness through social mobilisation programmes [13, 34]. Almost half (47.9%) of the adults had seen or heard message discouraging alcohol consumption while 18.7% had seen an advertisement promoting alcohol and 21.9% had attended social events, which exposed them to alcohol advertisements or offered free drinks [33]. Further intensifying anti-tobacco and anti-alcohol campaign can be useful in achieving and sustaining a faster decline in tobacco and alcohol consumption.

As per the PEN package, services like detection of hypertension, diabetes, assessment of cardiovascular disease risks, are envisioned to be available at health post level [13, 35]. It is imperative that the country focuses on the development and implementation of a community-based intervention for the prevention of metabolic risk factors. Nepal made significant achievements in reducing maternal, neonatal and child deaths through community-based interventions. Evidence suggests that similar intervention could be useful in the control of NCDs too [36]. Female Community Health Volunteers (FCHVs) involvement in the screening of some of the metabolic risk factors like raised blood pressure, high BMI among the high-risk population can be a cost-effective strategy [37] and could minimise the burden of exhausted healthcare workers. For regular monitoring of the cholesterol level and plasma glucose, FCHVs may refer to health facilities. Task sharing, particularly with other non-physician health workers, specifically in rural areas lacking an adequate number of physicians, could be an effective strategy for the management of NCDs [38].

Targeting intervention in hot spots and key populations affected by disease, scaling up intervention against major communicable diseases, integrating services wherever possible, identifying and implementing new and innovative approaches in disease prevention, and improving access could be potential strategies in dealing with CMNN diseases. Improving and maintaining a high level of vaccine coverage and intensifying health promotion activities could further reduce the burden. Care should be taken to make sure that CMNN diseases are not deprioritised while increasing focus on NCDs as it may lead to resurgence of the problems [39]. Broader social determinants of health like housing, income, and access to social supports can often serve as barriers at individual and community level to maintain optimum health. Addressing those broad determinants through appropriate public policies that promote health, strengthen community action and create an enabling environment can be effective [29].

As with the most forecasting studies, the findings have some limitations that arise from poor-quality and/or missing data. Data are from nationally representative studies are lacking on causes of disability, particularly low back pain and mental disorders. Nepal lacks a well-functioning vital registration to gather mortality data. However, even with these limitations, the estimates for Nepal are considered robust estimates of enough validity to support policymaking.

## Conclusions

Our analysis shows that by 2040 NCDs will be the leading cause of deaths in Nepal, with metabolic risk factors being the leading risk factors. An appropriate policy response will require significant investment to realign health sector priorities and resources, as they require extensive, multi-sectoral prevention measures, as well as long term and chronic care adjustments to health care services. Such re-allocation of resources will need a significant reorientation of the health system. The government of Nepal will need to respond to new paradigms of prevention programmes, as well as expanded programmes for chronic and long-term care, and significant re-alignments of financing.
